# CFTR Depletion Confers Hypersusceptibility to *Mycobacterium fortuitum* in a Zebrafish Model

**DOI:** 10.3389/fcimb.2020.00357

**Published:** 2020-07-17

**Authors:** Matt D. Johansen, Laurent Kremer

**Affiliations:** ^1^Institut de Recherche en Infectiologie de Montpellier, Centre National de la Recherche Scientifique UMR 9004, Université de Montpellier, Montpellier, France; ^2^INSERM, Institut de Recherche en Infectiologie de Montpellier, Montpellier, France

**Keywords:** *Mycobacterium fortuitum*, granuloma, infection, cording, cystic fibrosis, CFTR, pathogenesis, zebrafish

## Abstract

The *Mycobacterium fortuitum* complex comprises several closely related species, causing pulmonary and extra-pulmonary infections. However, there is very limited knowledge about the disease pathogenesis involved in *M. fortuitum* infections, particularly due to the lack of suitable animal models. Using the zebrafish model, we show that embryos are susceptible to *M. fortuitum* infection in a dose-dependent manner. Furthermore, zebrafish embryos form granulomas from as early as 2 days post-infection, recapitulating critical aspects of mycobacterial pathogenesis observed in other pathogenic species. The formation of extracellular cords in infected embryos highlights a previously unknown pathogenic feature of *M. fortuitum*. The formation of large corded structures occurs also during *in vitro* growth, suggesting that this is not a host-adapted stress mechanism deployed during infection. Moreover, transient macrophage depletion led to rapid embryo death with increased extracellular cords, indicating that macrophages are essential determinants of *M. fortuitum* infection control. Importantly, morpholino depletion of the cystic fibrosis transmembrane conductance regulator (*cftr*) significantly increased embryo death, bacterial burden, bacterial cords and abscesses. There was a noticeable decrease in the number of *cftr*-deficient infected embryos with granulomas as compared to infected controls, suggesting that loss of CFTR leads to impaired host immune responses and confers hypersusceptiblity to *M. fortuitum* infection. Overall, these findings highlight the application of the zebrafish embryo to study *M. fortuitum* and emphasizes previously unexplored aspects of disease pathogenesis of this significant mycobacterial species.

## Introduction

The incidence of non-tuberculous mycobacterial (NTM) infections is increasing globally, surpassing the infection rate of tuberculosis in many developed countries (Johansen et al., 2020). NTM are increasingly acknowledged pathogens capable of infecting a vast array of tissues and inducing a wide spectrum of clinical symptoms in humans, with pulmonary infections being the most common clinical manifestations (Ratnatunga et al., 2020). NTM infections are particularly prevalent in immunocompromised individuals and those with underlying genetic and structural defects. They have emerged in recent years as prevalent pathogens in cystic fibrosis (CF) patients, with several global CF centers reporting the isolation of NTM in the respiratory tract of CF patients (Roux et al., 2009; Skolnik et al., 2016). NTM prevalence in CF patients varies between 5 and 20%, with the *Mycobacterium avium* complex and the *Mycobacterium abscessus* complex being the most significant species (Richards and Olivier, 2019). However, less frequently isolated species in CF patients include other NTM, such as *Mycobacterium gordonae, Mycobacterium kansasii* or *M. fortuitum* (Sermet-Gaudelus et al., 2003; Cândido et al., 2014; Martiniano et al., 2016; Richards and Olivier, 2019).

The *M. fortuitum* complex includes *M. fortuitum, Mycobacterium peregrinum, Mycobacterium porcinum, Mycobacterium septicum, Mycobacterium conceptionense, Mycobacterium boenickei, Mycobacterium houstonense, Mycobacterium neworleansense, Mycobacterium brisbanense, Mycobacterium farcinogenes, Mycobacterium senegalense*, and *Mycobacterium setense* (Brown-Elliott and Wallace, 2002; Brown-Elliott and Philley, 2017; Tortoli et al., 2017). *M. fortuitum* is a rapid-growing and frequently identified NTM species, causing localized skin and soft tissue infections (Wallace et al., 1983). Several clinical cases have also reported catheter infections, post-surgical infections, peritonitis, eye infections, and pulmonary infections (Brown-Elliott et al., 2012; Brown-Elliott and Philley, 2017). Unfortunately, our understanding surrounding the pathogenicity of *M. fortuitum* has been hampered by the lack of genetic tools and by the restricted panel of cellular and animal models available. Among the few animal models reported, a murine infection model was characterized by a sustained persistent infection of *M. fortuitum* in the kidneys (Parti et al., 2005, 2008). However, while the bacilli proliferated freely inside murine macrophages they did not invade a murine kidney cell line (Parti et al., 2005). A murine neutropenic model, in which neutropenia is induced by intraperitoneal doses of cyclophosphamide, has also been exploited to demonstrate the *in vivo* efficacy of drugs against *M. fortuitum* and *M. abscessus* (Das et al., 2019). In addition, a recent study highlighted the suitability of the *Galleria mellonella* moth larvae as a cheap, efficient and rapid *in vivo* model for the screening of antibiotic combinations and novel treatments against NTM, including *M. fortuitum* (Entwistle and Coote, 2018; García-Coca et al., 2019).

Infection of mice with *M. fortuitum* are also accompanied by visible symptoms, such as spinning disease (Saito and Tasaka, 1969; Parti et al., 2005), often associated with neurological disorders. Interestingly, spinning disease due to invasive abscess formation within the central nervous system has been described both in mice as well as in zebrafish embryos infected with *M. abscessus* (Saito and Tasaka, 1969; Bernut et al., 2014a), suggesting that both *M. abscessus* and *M. fortuitum* display common pathogenic traits in these animals. Interestingly, *M. fortuitum*, formerly *Mycobacterium ranae*, was originally recovered from frogs in 1905 and considered a pathogen for animals and humans since its first isolation from a human abscess in 1938. Together with *Mycobacterium marinum*, it is commonly associated with fish tuberculosis, a systemic and chronic disease characterized by the presence of granulomatous reactions. Supporting the view that *M. fortuitum* infects amphibians and fish, inoculation in adult goldfish led to the development of a characteristic chronic granulomatous response similar to that associated with natural mycobacterial infection (Talaat et al., 1999). Overall, these observations suggest that zebrafish may represent a valuable model to study pathogenesis of this understudied mycobacterial species.

Zebrafish have recently gained favor as a useful and amenable model to study host-bacterial interactions (Davis et al., 2002; van der Sar et al., 2004; Clay et al., 2007; Prajsnar et al., 2008, 2013; Vergunst et al., 2010; Alibaud et al., 2011; Cambier et al., 2014; Gomes and Mostowy, 2020). Due to genetic tractability and optical transparency, zebrafish embryos represent an exquisite model to study important aspects of infectious diseases. Whilst the adult zebrafish possess a complex immune system similar to that of humans, comprising both the innate and adaptive arms of immunity, the early embryonic stages solely harbor innate immunity (Davis et al., 2002). Infection of embryos, thus allows a more in-depth focus on the role of innate immunity during the very early stages of infection (Davis et al., 2002; Torraca and Mostowy, 2018). Zebrafish infection with the fish pathogen *M. marinum* has led to remarkable insights into the understanding of human tuberculosis (Davis et al., 2002; Berg and Ramakrishnan, 2012), the role of macrophages in pathogen dissemination (Davis and Ramakrishnan, 2009), infection-induced antibiotic tolerance (Adams et al., 2011), the contribution of ESX secretion system in granuloma formation (Volkman et al., 2004) and induced granuloma-associated angiogenesis which promotes mycobacterial growth and facilitates the spread of infection to distant tissue sites (Oehlers et al., 2015). However, a noticeably important breakthrough came from the *M. abscessus* model of infection in zebrafish, which unraveled the importance of cording as mechanism of immune evasion (Bernut et al., 2014a) and the role of TNF signaling in controlling infection (Bernut et al., 2016a). Because many conserved virulence mechanisms and host susceptibility determinants identified during zebrafish infection have been validated in humans (Tobin et al., 2010; Bernut et al., 2019), we reasoned that zebrafish may also represent a useful experimental model to decipher the virulence and immunopathology of *M. fortuitum* infections.

Herein, we have exploited the zebrafish embryo as an amenable model for the study of systemic *M. fortuitum* infections. We describe the aggressive and lethal infections caused by *M. fortuitum*, which develops in the absence of either functional innate immunity or CFTR.

## Materials and Methods

### Mycobacterial Strains and Culture Conditions

*Mycobacterium abscessus* CIP104536^T^, *M. fortuitum* subsp. *fortuitum* (ATCC 6841) and *Mycobacterium smegmatis* mc^2^155 were routinely grown and maintained at 37°C in Middlebrook 7H9 broth (BD Difco) supplemented with 10% oleic acid, albumin, dextrose, catalase (OADC; BD Difco) and 0.025% Tyloxapol (Sigma-Aldrich) (7H9^OADC/T^) or on Middlebrook 7H10 supplemented with 10% OADC enrichment (7H10^OADC^) and in the presence of antibiotics if required. To observe *in vitro* liquid growth phenotypes, bacteria were grown in Cation-adjusted Mueller-Hinton Broth (CaMHB; Sigma-Aldrich).

### Creation of Fluorescent *M. fortuitum*

Fluorescent *M. fortuitum* was generated using the pVV16-eGFP replicative vector (Vilchèze et al., 2014). Electro-competent *M. fortuitum* were generated as previously described (Viljoen et al., 2018). *M. fortuitum* was placed on ice for 2 h prior to pelleting by centrifugation (3,000 × g at 4°C for 15 min). Following centrifugation, bacteria were resuspended in decreasing volumes of wash buffer (10% glycerol (v/v) and 0.025% Tyloxapol in distilled water) and pelleted by centrifugation for a total of 4 washes. For electroporation transformation, 1 μg of plasmid DNA was added to 200 μL of electrocompetent bacteria and transferred to a chilled 0.2 cm electrode gap GenePulser electroporation cuvette (Bio-rad) and transformed using a GenePulser Cxell electroporator (Bio-rad) (2.5 kV, 1, 000 Ω and 25 μF). Bacteria were recovered in 800 μL of 7H9^OADC/T^ and placed at 37°C overnight. For selection of green fluorescent colonies, *M. fortuitum* was plated on 7H10^OADC^ supplemented 50 μg/mL kanamycin (Euromedex). Positive colonies were selected based on fluorescence and maintained in 7H9^OADC/T^ supplemented with 50 μg/mL kanamycin. Fluorescent *M. abscessus* has been previously described (Bernut et al., 2014a).

### Preparation of Single Cell *M. fortuitum*

Single cell preparations of fluorescent *M. fortuitum* were created, as previously described (Bernut et al., 2015). Exponential-phase *M. fortuitum* were pelleted at 3000 × g for 10 min at room temperature and resuspended in 1 mL of 7H9^OADC/T^. Bacteria were passed through a 26 gauge needle 15 times, followed by two rounds of 10 s sonication, made up to 50 mL with 7H9^OADC/T^ and centrifuged at 3000 × g for 10 min at room temperature. Pelleted bacteria were resuspended in up to 1 mL of 7H9^OADC/T^ and 10 μL aliquots of single cell *M. fortuitum* prepared and placed at −80°C until required.

### Zebrafish Maintenance

Zebrafish experiments were completed in accordance with the Comité d'Ethique pour l'Expérimentation Animale de la Région Languedoc Roussillon under the reference CEEALR36-1145. All experiments in the current study were performed using the *golden* mutant and macrophage reporter Tg(*mpeg1:mCherry)* lines as previously described (Bernut et al., 2014a, 2015). Zebrafish embryos were obtained and maintained as previously described (Bernut et al., 2015).

### Morpholino Injection and *cftr* Knockdown

Morpholinos were designed and purchased from GeneTools. A splice-blocking morpholino specifically targeting zebrafish gene *cftr* (ZEBRAFISHIN, ZDB-GENE-050517-20) (5′-GACACATTTTGGACACTCACACCAA-3′) was injected into zebrafish embryos at the 1–4 cell stage (1 mM, 2 nL). Furthermore, details of the morpholino efficacy and specificity and *cftr* knockdown was previously validated as described (Bernut et al., 2019). Briefly, this splice blocking morpholino was designed against the exon 3-intron 3 junction within the *cftr* gene. Sequencing analysis indicated that the *cftr* morpholino blocks normal splicing, resulting in a 54 bp deletion in exon 3, and leading to the knockdown of CFTR expression (Bernut et al., 2019).

### Zebrafish Microinjection and Infection

At 24 h post-fertilization, embryos were dechorionated using Pronase (10 mg/mL; Sigma-Aldrich) for up to 5 min at room temperature, followed by extensive washing in zebrafish water. Following dechorionation, embryos were injected with either liposomal clodronate or PBS-filled liposomes (Liposoma) (2 nL) *via* caudal vein injection, as previously described (Bernut et al., 2014a, 2015). At 30 h post-fertilization, embryos were anesthetized in 0.02% tricaine solution and infected with fluorescent *M. fortuitum via* caudal vein injection (3 nL containing ≈100 bacteria/nL). Bacterial inoculum was checked *a posteriori* by injection of 3 nL into sterile PBS and plating onto 7H10^OADC^. Following infection, embryos were transferred to 24-well plates (2 embryos/well) and incubated at 28.5°C for the duration of the experiment. Embryo age is expressed as days post-infection (dpi).

### Zebrafish Monitoring and Live Imaging

Embryo survival was monitored daily based on the presence or absence of a heartbeat. Survival curves were determined by counting dead larvae for up to 12 days, or until uninfected embryos begin to die. At designated key time points post-infection, embryos were anesthetized in 0.02% tricaine solution and mounted on 3% (w/v) methylcellulose solution for live imaging. Images were taken using a Zeiss Axio Zoom.V16 coupled with an Axiocam 503 monochrome camera (Zeiss). Fluorescent Pixel Count (FPC) measurements were determined using the “Analyse particles” function in ImageJ. Granulomas were identified based on the co-localization of fluorescent macrophages and fluorescent *M. fortuitum*. All experiments were completed at least two times independently.

### Statistical Analysis

Survival curve analysis was completed using the log-rank (Mantel-Cox) statistical test. Abscess, cord, granuloma and bacterial burden (FPC) analyses were completed using unpaired Student′s *t*-test. All statistical tests were completed using Graphpad Prism (Version 8.0.1).

## Results

### *M. fortuitum* Forms Large Corded Aggregates *in vitro*

**Figure 1:**
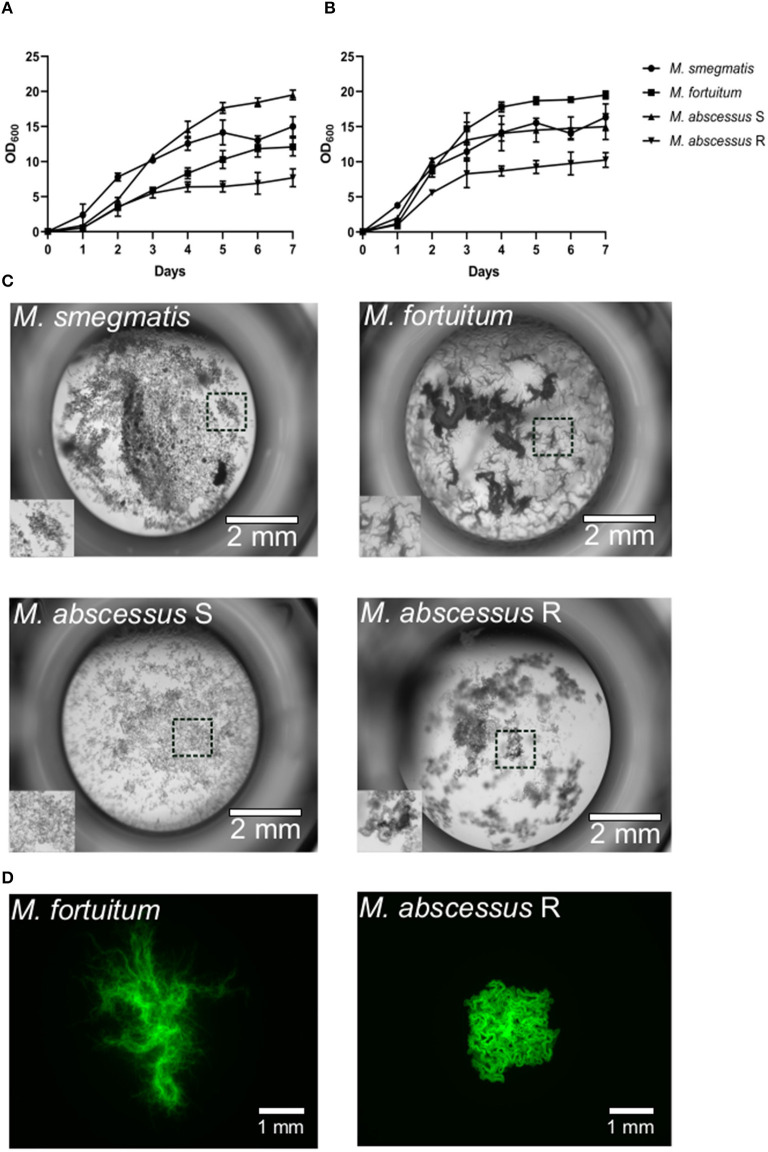
*Mycobacterium fortuitum* forms large corded bacterial aggregates *in vitro*. **(A)** *M. abscessus* CIP104536^T^ smooth (S) and rough (R) variants, *M. fortuitum* subsp. *fortuitum* (ATCC 6841) and *M. smegmatis* mc^2^155 were inoculated in 7H9^OADC/T^ at an optical density of 0.05 (OD_620_) and incubated at 30°C and **(B)** 37°C under shaking at 100 rpm. Growth measurements were taken daily over a 7-day period until cultures reached stationary phase. Data shown is the merge of two independent experiments. Error bars represent standard deviation. **(C)** *In vitro* liquid growth properties of the different NTM species in CaMHB medium following 3–4 days static culture at 30°C in a 96-well plate. The boxed area depicts the zoomed section of liquid growth in the bottom left hand corner of each well. Scale bars represent 2 mm. **(D)** *In vitro* solid agar growth properties of *M. fortuitum* and *M. abscessus* R variant following 3 days growth at 30°C. Scale bars represent 1 mm.

Zebrafish naturally require lower ambient temperatures of ~28°C for normal growth and development. In establishing a new model to study the pathogenesis of a disease-causing agent, it is critical that the replication of the pathogen is not affected by these temperatures. As has been reported for other NTM such as *M. abscessus* (Bernut et al., 2014a) and *M. kansasii* (Johansen and Kremer, 2020), these species are able to replicate at lower ambient temperatures, albeit at a slower rate, highlighting their adaptability to the zebrafish platform. We firstly wanted to determine whether *M. fortuitum* is able to grow at lower temperatures compared to traditional 37°C incubation. When grown in 7H9^OADC/T^ at 30°C, we observed that *M. fortuitum* was able to reach early stationary phase around Day six, which was comparable to other rapid growers that are known to thrive at lower temperatures, such as *M. abscessus* and *M. smegmatis* (Figure 1A). Comparatively, all species grew at a much faster rate and reached stationary phase earlier when grown at 37°C, which was to be expected and has been reported for other NTM species (Johansen and Kremer, 2020) (Figure 1B).

It has been well-described that specific mycobacteria are able to grow in large cord-like aggregates; a trait that has been often regarded for the most pathogenic mycobacteria such as *Mycobacterium tuberculosis, M. marinum, M. abscessus* and recently *M. kansasii* (Glickman et al., 2000; Staropoli and Branda, 2008; Bernut et al., 2014a, 2016b; Johansen and Kremer, 2020). As such, we wanted to determine whether *M. fortuitum* also possessed similar traits, which may explain why it is regarded as an opportunistic pathogen and frequently isolated in pulmonary and extra-pulmonary sites (Wallace et al., 1983; Brown-Elliott and Philley, 2017). Remarkably, when grown at both 30 and 37°C in CaMHB, a liquid medium commonly used for testing of antimicrobial susceptibility, we observed the formation of large cord-like aggregates for *M. fortuitum* (Figure 1C). These cords were different in size and appearance when compared to rough *M. abscessus*; an NTM species well-known for its large extracellular cord formation both *in vitro* and *in vivo* (Bernut et al., 2014a, 2016a,b). When we observed bacterial growth on 7H10^OADC^ solid agar, we also identified the presence of large cord-like structures for *M. fortuitum*, which were noticeably different in appearance to rough *M. abscessus* (Figure 1D).

### *M. fortuitum* Is Pathogenic in a Zebrafish Model in a Dose-Dependent Manner

**Figure 2:**
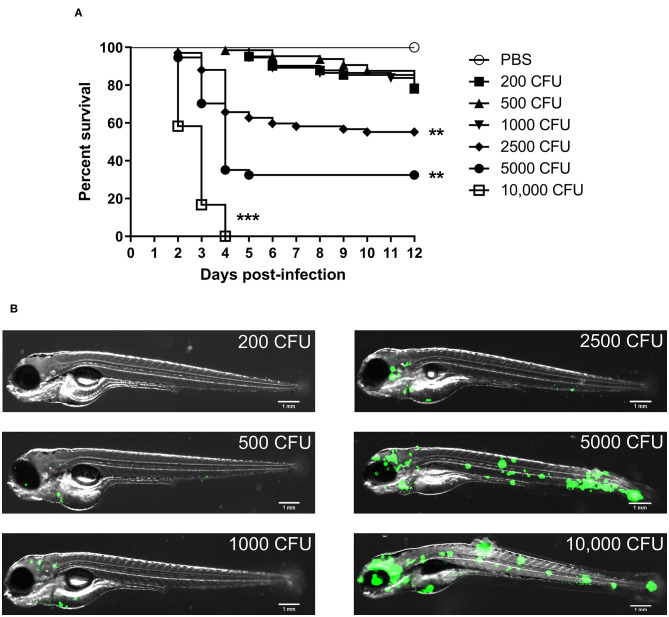
Zebrafish embryos are susceptible to *Mycobacterium fortuitum* infection in a dose-dependent manner. **(A)** Zebrafish embryos were injected with varying dosages of GFP-expressing *M. fortuitum* at 30 h post-fertilization *via* caudal vein injection. Embryo survival was monitored over a 12 day period. *N* = 20–25 embryos/group. Statistical analysis was completed using the log-rank (Mantel-Cox) statistical test for survival curves. Data shown is the merge of two independent experiments. **(B)** Representative images of zebrafish embryos infected with varying dosages of GFP-expressing *M. fortuitum* at 2 days post-infection. Green represents *M. fortuitum*. Scale bars represent 1 mm. **P ≤ 0.01, ***P ≤ 0.001.

We firstly wanted to determine whether *M. fortuitum* was able to promote embryonic death in a dose-dependent manner. At 30 h post-fertilization, bacterial suspensions of *M. fortuitum* were injected in the caudal vein of zebrafish embryos. We observed that embryos infected with 200, 500 or 1,000 CFU of *M. fortuitum* induced ~20% embryonic death in a 12-day period (Figure 2A). This is similar to what has been observed for other NTM, such as the smooth variant of *M. abscessus* (Bernut et al., 2014a) or *M. kansasii* (Johansen and Kremer, 2020). Moreover, infection with higher doses, corresponding to 2,500 or 5,000 CFU, led to 40 and 70% of embryo death, respectively, within a 12-day period. Interestingly, larval killing occurred rapidly (within 4 days post-infection) after which the percentage of survival remained stable. Embryos infected with 10,000 CFU all succumbed within a 4-day period. Whole embryo imaging demonstrates that larval killing was directly correlated to the bacterial burden and that bacteria were disseminated throughout the entire embryo, suggestive of a systemic infection (Figure 2B). Collectively, these findings demonstrate the feasibility of the zebrafish model to study acute infections with *M. fortuitum*.

### The Zebrafish*/M. fortuitum* Model Recapitulates Important Mycobacterial Pathophysiological Features

**Figure 3:**
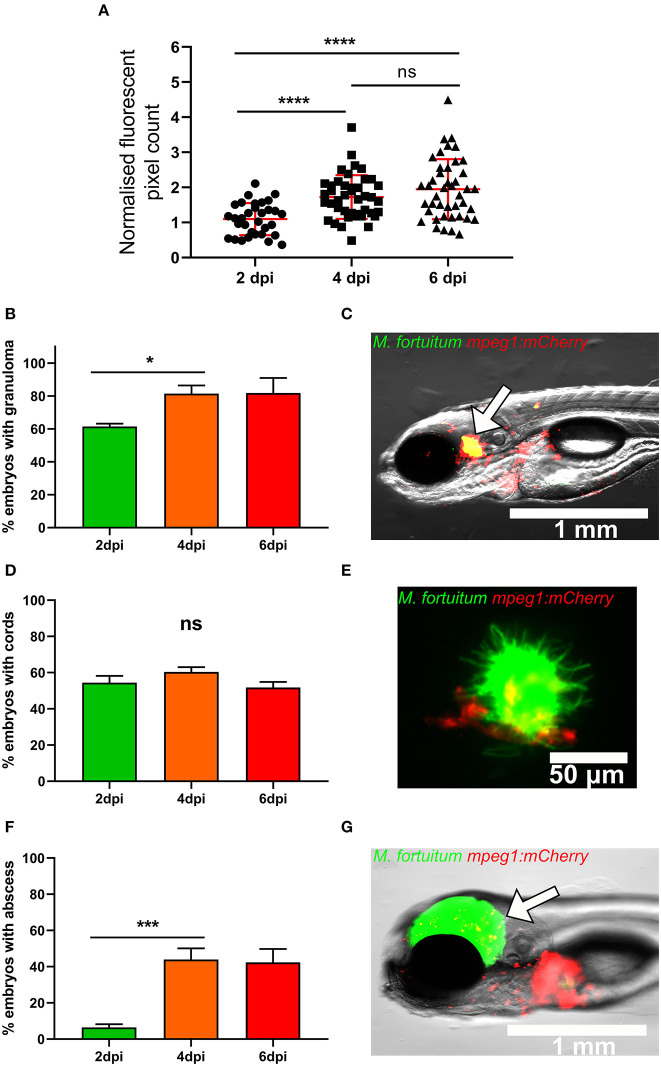
Zebrafish infected with *Mycobacterium fortuitum* recapitulate the hallmark features of other pathogenic mycobacteria. **(A)** Following infection *mpeg1::mCherry* zebrafish embryo at 30 h post-fertilization *via* caudal vein injection with 300 CFU of GFP-expressing *M. fortuitum*, bacterial burden was quantified using fluorescent pixel count (FPC determination using ImageJ software) at 2, 4, and 6 days post-infection. For each experiment, all groups were normalized against the 2 days post-infection group. Each datapoint represents an individual embryo. Error bars represent standard deviation. Statistical significance was determined by Student′s *t*-test. Plots represent a pool of 2 independent experiments containing approximately 20 embryos per group. **(B)** The granuloma formation kinetic in embryos following infection with *M. fortuitum*. Error bars represent standard deviation. Data shown is the merge of two independent experiments. Statistical significance was determined by Student′s *t*-test. **(C)** Representative image of a granuloma at 4 days post-infection. The white arrow highlights the granuloma. Scale bar represents 1 mm. **(D)** The kinetic of bacterial cord formation in zebrafish embryos following *M. fortuitum* infection. Error bars represent standard deviation. Data shown is the merge of two independent experiments. Statistical significance was determined by Student′s *t*-test. **(E)** Representative image of a bacterial corded *M. fortuitum* aggregate surrounded by recruited macrophages at 4 days post-infection. Note the sheer size of the bacterial aggregate in comparison to the smaller macrophages. Green represents *M. fortuitum*, while red represents macrophages. Scale bar represents 50 μm. **(F)** The kinetic of abscess formation in zebrafish embryos infected with *M. fortuitum*. Error bars represent standard deviation. Data shown is the merge of two independent experiments. Statistical significance was determined by Student′s *t*-test. **(G)** Representative image of an *M. fortuitum* abscess at 4 days post-infection. The white arrow highlights the abscess. Green represents *M. fortuitum*, while red represents macrophages. Scale bar represents 1 mm. *P ≤ 0.05, ***P ≤ 0.001, ****P ≤ 0.0001.

We next monitored the kinetic of the bacterial burden upon intravenous infection of embryos with 300 CFU of *M. fortuitum* expressing GFP by determination of fluorescent pixel counts (FPC). There was a significant increase in the bacterial loads between 2 and 4 dpi, after which bacterial loads remained stable between 4 and 6 dpi, suggesting that the bacterial expansion is controlled at later time points (Figure 3A).

A major feature of mycobacterial infection is the formation of the granuloma (Pagán and Ramakrishnan, 2018). Importantly, zebrafish embryos do not possess adaptive immunity and so granuloma identification is strictly based on the presence of macrophages at the infection foci (Davis et al., 2002). This host immune aggregate acts to restrict pathogen spread, however mycobacteria have developed unique mechanisms allowing their prolonged survival within these host structures (Volkman et al., 2004; Davis and Ramakrishnan, 2009; Bernut et al., 2014a; Johansen and Kremer, 2020). Comparatively, extracellular cord formation within the zebrafish embryo is often representative of acute infection and generally is indicative of mycobacterial escape from the phagosome and macrophage destruction (Bernut et al., 2014a, 2015, 2016a). Abscess formation often represents loss of infection control and typically occurs following extracellular cord formation and expansion (Bernut et al., 2014a, 2015, 2016a). Abscesses represent a marker of disease severity and are often associated with cellular debris, tissue destruction, and acute infection in zebrafish (Bernut et al., 2014a). As such, we wanted to determine whether zebrafish embryos infected with *M. fortuitum* are able to recapitulate critical determinants of mycobacterial infection. To visualize and monitor the presence of granulomas, infections with *M. fortuitum* expressing GFP were carried out in the caudal vein of the *mpeg1:mCherry* zebrafish reporter line harboring red fluorescent macrophages. We observed the presence of granulomas in approximately 60% of infected embryos at 2 dpi, which increased to 80% of embryos by 4 dpi and then remained constant between 4 and 6 dpi (Figures 3B,C). This coincides with the arrest of bacterial proliferation between 4 and 6 dpi as seen in Figure 3A. When we counted the number of embryos with cords, ~50% of embryos displayed these extracellular bacterial structures, which was consistent throughout the experiment until 6 dpi (Figures 3D,E). We observed a significant increase in the number of embryos with abscesses from 5 to 40% between 2 and 4 dpi (Figures 3F,G). These findings demonstrate that the zebrafish model effectively recapitulates critical aspects of mycobacterial pathophysiology following *M. fortuitum* infection and highlights the applicability of this model for the study of *M. fortuitum* pathogenesis. These results also suggest that acute infection is typified by rapid bacterial expansion and that cord formation occurs very rapidly within 2 dpi, while the bacterial growth is controlled once granulomas are fully formed at 4 dpi.

### Macrophages Are Essential Host Determinants of *M. fortuitum* Infection Control

**Figure 4:**
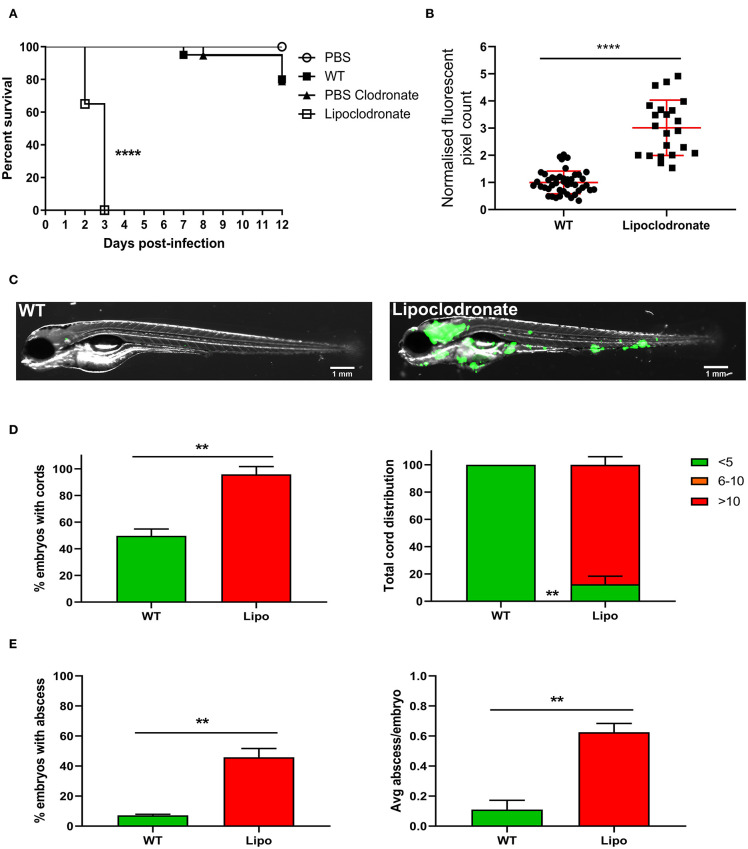
Lipoclodronate macrophage depletion results in lethal *M. fortuitum* infection. **(A)** At 24 h post-fertilization, embryos were treated with liposomal clodronate *via* caudal vein injection to transiently deplete macrophages. At 30 h post-fertilization, embryos were injected intravenously with 300 CFU of GFP-expressing *M. fortuitum* with embryo survival monitored over a 12 day period. *n* = 20–25 embryos/group. Statistical analysis was completed using the log-rank (Mantel-Cox) statistical test for survival curves. Data shown is the merge of two independent experiments. **(B)** Bacterial burden at 2 days post-infection was calculated using fluorescent pixel count (FPC) determination with ImageJ software. Lipoclodronate-treated embryos were normalized against corresponding controls in each experiment. Each datapoint represents an individual embryo. Error bars represent standard deviation. Statistical significance was determined by Student′s *t*-test. Plots represent a pool of 2 independent experiments containing approximately 20 embryos per group. **(C)** Representative images of wild-type (WT) and Lipoclodronate-treated embryos infected with 300 CFU of GFP-expressing *M. fortuitum* at 2 days post-infection. Scale bars represent 1 mm. **(D)** The proportion of embryos with bacterial cords and the total distribution of cords categorized as low (<5 cords/embryo), moderate (6–10 cords/embryo) and high (>10 cords/embryo) in *M. fortuitum*-infected embryo at 2 days post-infection. Error bars represent standard deviation. Data shown is the merge of two independent experiments. Statistical significance was determined by Student′s *t*-test. **(E)** The proportion of embryos with abscesses and the average number of abscesses per infected embryo at 2 days post-infection. Error bars represent standard deviation. Data shown is the merge of two independent experiments. Statistical significance was determined by Student′s *t*-test. **P ≤ 0.01, ****P ≤ 0.0001.

Macrophages are the first responders to infection and one of the major cell populations within the mycobacterial granuloma (Volkman et al., 2004; Bernut et al., 2016a; Pagán and Ramakrishnan, 2018). It is well-known that macrophage depletion has a detrimental outcome for mycobacterial infection in zebrafish embryos, leading to premature larval death within several days post-infection (Clay et al., 2007; Bernut et al., 2014a). Currently, it is not known whether macrophages play such a crucial role in *M. fortuitum* infection as compared to other mycobacterial species. As such, we aimed to elucidate whether macrophages are essential determinants of *M. fortuitum* infection control. Liposomal clodronate is highly effective in depleting macrophages within the zebrafish embryo for up to 6 days as previously reported (Bernut et al., 2014a, 2015). Using Lipoclodronate macrophage depletion, 100% killing was achieved in animals infected with ≈300 CFU at 3 dpi (Figure 4A). This was associated with a massive increase in bacterial loads in macrophage-depleted embryos as compared to wild-type fish receiving PBS clodronate at 2 dpi, thus highlighting the critical role of macrophages in controlling *M. fortuitum* infection (Figure 4B). Further, embryo imaging clearly highlighted the very high bacterial loads in the macrophage-depleted embryos as compared to the wild-type fish (Figure 4C), consistent with bacterial burden quantification. Importantly, we observed a greater proportion of embryos with cords and an increase in the number of cords per embryo in Lipoclodronate-treated embryos as compared to PBS-injected animals, emphasizing the need of macrophages to restrict intracellular growth and preventing extracellular cord formation (Figure 4D). There was also a greater proportion of Lipoclodronate treated embryos with abscesses and a higher number of abscesses per embryo when compared to PBS groups at 2 dpi (Figure 4E). Together, these results demonstrate that the hypersusceptibility of Lipoclodronate-treated embryos to *M. fortuitum* stems from their lack of macrophages.

### CFTR Depletion Leads to Increased Susceptibility to *M. fortuitum* Infection

**Figure 5:**
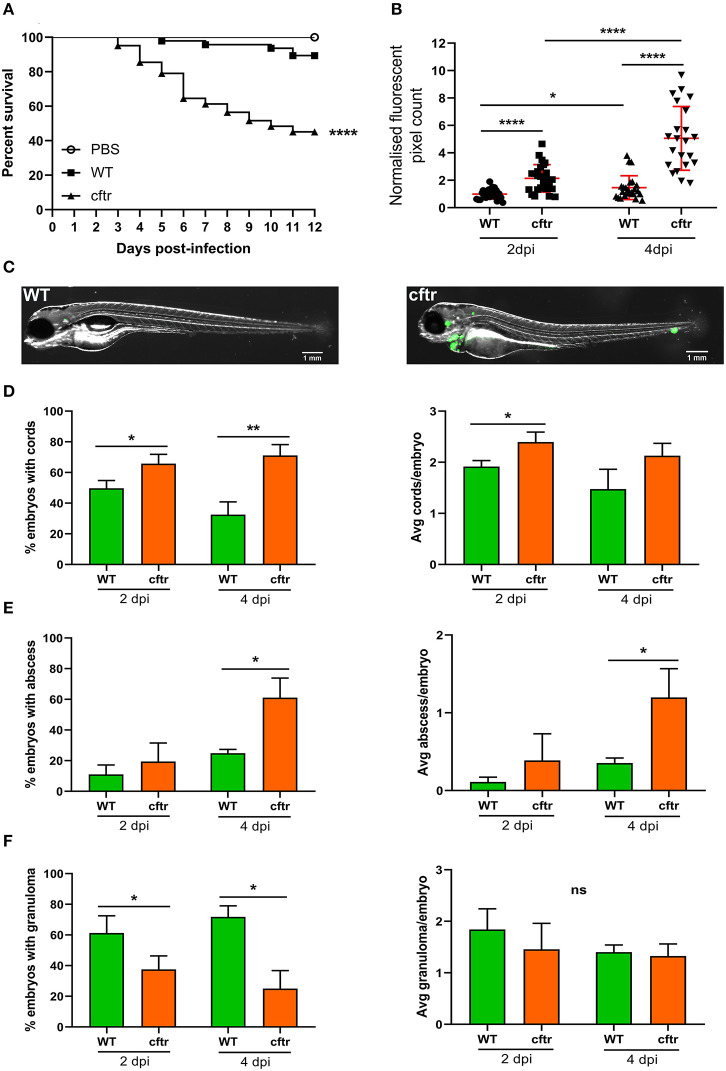
CFTR ablation leads to rapid larval death and uncontrolled bacterial expansion. **(A)** *Cftr* mrophants at 30 h post-fertilization were infected with 300 CFU of GFP-expressing *M. fortuitum via* caudal vein injection and embryo survival was monitored over a 12 day period. *N* = 20–25 embryos/group. Statistical analysis was completed using the log-rank (Mantel-Cox) statistical test for survival curves. Data shown is the merge of three independent experiments. **(B)** Bacterial burden was quantified by fluorescent pixel count (FPC) determination using ImageJ software, with each data point representing an individual embryo. Each group was normalized against the wild-type cohort at 2 days post-infection. Error bars represent standard deviation. Statistical significance was determined by Student′s *t*-test. Plots represent a pool of three independent experiments containing ~20–25 embryos per group. **(C)** Representative images of wild-type (WT) and CFTR depleted (*cftr*) embryos infected with 300 CFU of GFP-expressing *M. fortuitum* at 2 days post-infection. Scale bars represent 1 mm. **(D)** The proportion of embryos with bacterial cords and the average number of cords per infected embryo at 2 and 4-days post-infection. Error bars represent standard deviation. Data shown is the merge of three independent experiments. Statistical significance was determined by Student′s *t*-test. **(E)** The proportion of embryos with abscesses and the average number of abscesses per infected embryo at 2 and 4-days post-infection. Error bars represent standard deviation. Data shown is the merge of three independent experiments. Statistical significance was determined by Student′s *t*-test. **(F)** The proportion of embryos with granulomas and the average number of granulomas per infected embryo at 2 and 4-days post-infection. Error bars represent standard deviation. Data shown is the merge of three independent experiments. Statistical significance was determined by Student′s *t*-test. *P ≤ 0.05, **P ≤ 0.01, ****P ≤ 0.0001.

It is estimated that 5–20% of CF patients will develop NTM infection (Richards and Olivier, 2019), indicating that these patients are at increased risk of infection due to genetic susceptibility. Although less frequently encountered than *M. avium* or *M. abscessus*, several studies have reported the presence of *M. fortuitum* in CF patients (Sermet-Gaudelus et al., 2003; Cândido et al., 2014; Martiniano et al., 2016; Richards and Olivier, 2019). Recent work has shown that CFTR is important for fine-tuning host oxidative stress and restricting intracellular growth of *M. abscessus* in a zebrafish model (Bernut et al., 2019). These embryos also possessed fewer granulomas and granulomas displayed a disorganized structure, suggesting that CFTR is also an important determinant of infection containment within the granuloma microenvironment. We thus inquired whether CFTR depletion also conferred increased susceptibility to *M. fortuitum* infection. Using a morpholino knockdown strategy previously validated to target the zebrafish *cftr* gene (Bernut et al., 2019), we observed a massive increase in embryo mortality (almost 60% mortality at 12 dpi) following *M. fortuitum* infection in *cftr* morphants when compared to wild-type infected embryos (Figure 5A). In addition, mortality started earlier in *cftr* morphants than in wild-type fish. When we further examined bacterial burden, we observed an increase in CFTR-depleted embryos at 2 dpi when compared to wild-type infection, which was greatly increased by 4 dpi (Figures 5B,C). Strikingly, in CFTR-depleted embryos, there was an enhanced proportion of embryos with cords at both 2 and 4 dpi when compared to wild-type infection. Moreover, there was an increase in the number of cords per embryo in CFTR-depleted embryos at 2 dpi when compared to wild-type (Figure 5D). There was also a significantly greater number of embryos with abscesses and a greater number of abscesses per embryo at 4dpi in the CFTR-depleted group as compared to wild-type infections (Figure 5E). In contrast, infected *cftr* morphants produced less granulomas when compared to wild-type infections, however the number of granulomas per embryo remained constant over time (Figure 5F), similarly to what was previous observed in *M. abscessus* infected *cftr* morphants (Bernut et al., 2019). In contrast to wild-type embryos where bacterial proliferation is constrained when granulomas are formed (Figure 3), the opposite scenario occurs in *cftr* morphants whereby bacterial proliferation progresses whilst the number of granulomas remains unchanged. These findings suggest that depletion of CFTR impairs host immune responses resulting in granuloma formation.

Overall, these findings emphasize the importance of CFTR as a host factor in controlling *M. fortuitum* pathogenesis through granuloma formation and preventing extracellular cord formation, indicating that CFTR dysfunction results in hypersusceptibility to *M. fortuitum* infection.

## Discussion

**Figure 6:**
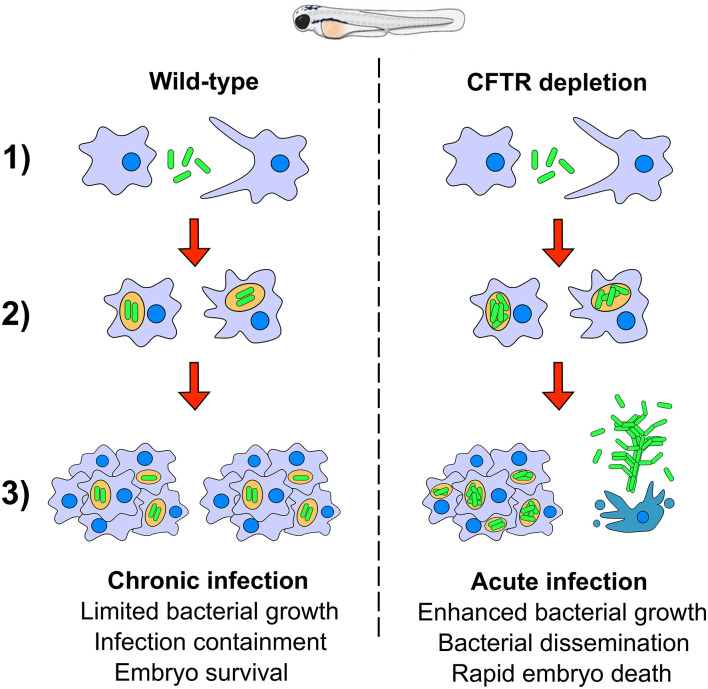
A hypothetical schematic summarizing the pathogenesis of *Mycobacterium fortuitum* in both wild-type and CFTR backgrounds. (1) Following intravenous injection of *M. fortuitum* into zebrafish embryos, macrophages are rapidly recruited to the site of infection and phagocytose bacilli; (2) Once inside the phagosome, *M. fortuitum* is able to initially replicate, however in a wild-type scenario, host defenses are able to slow the bacterial expansion until further macrophages are recruited. Comparatively, following CFTR depletion, bacteria are able to rapidly expand within the phagosome, presumably due to defective host oxidative defense mechanisms. (3) Additional macrophages are recruited to the infection foci, forming the granuloma which acts to contain infection dissemination in wild-type embryos. In a proportion of wild-type embryos, we propose that macrophage apoptosis triggers the escape of bacilli to the extracellular space which facilitates the growth of bacilli into large corded aggregates, promoting zebrafish embryo death. In CFTR-depleted embryos, there are fewer granulomas and a greater proportion of cords which leads to more rapid embryonic death and a greater proportion of cords and abscesses.

*Mycobacterium fortuitum* is increasingly recognized as an opportunistic nosocomial pathogen responsible for a wide panel of clinical infections, including lung diseases. However, our understanding of the pathogenic mechanisms of this important NTM species remains obscured by the lack of relevant animal models. Herein, we exploited the zebrafish model to directly and non-invasively dissect critical steps in the pathogenesis of *M. fortuitum*. Genetic manipulation of the host, combined with a fluorescent reporter bacterial strain were used to decipher the interplay between *M. fortuitum* and host macrophages. We provide evidence that zebrafish embryos, displaying only innate immunity, represent a suitable and permissive host that is susceptible to high doses of *M. fortuitum*. Chemical depletion of macrophages led to a rapid expansion of *M. fortuitum*, establishing a systemic and acute infection leading to rapid larval killing. Most importantly, morpholino depletion of CFTR led to hypersusceptibility of zebrafish embryos to *M. fortuitum* infection, providing the first insight into the significance of CFTR in *M. fortuitum* pathogenesis. The pathophysiological events of *M. fortuitum* infection are reminiscent to those reported earlier for *M. abscessus*, a well-acknowledged CF pathogen and can be summarized in a sequential manner (Figure 6): (1) Following intravenous injection, *M. fortuitum* is rapidly phagocytosed by macrophages; (2) Once inside the phagosome, *M. fortuitum* is initially able to replicate until the recruitment of additional macrophages to the site of infection; (3) Infection is contained with the formation of the mycobacterial granuloma and leads to a chronic infection. However, in a subset of wild-type embryos and to a much greater extent in CFTR embryos, it is very likely that the escape or release of *M. fortuitum* from infected macrophages is favoring extracellular bacterial multiplication in the form or serpentine cords (highly stimulated in macrophage-depleted embryos). Bacterial cords can withstand phagocytosis by macrophages which further exacerbates uncontrolled bacterial multiplication and ultimately results in abscess formation, tissue damage and larval death. This is supported by recent findings indicating that caspase-mediated apoptosis of catfish macrophages occurs rapidly after infection with *M. fortuitum* (Datta et al., 2018). Thus, one can envisage a similar scenario where apoptosis of zebrafish macrophages releases *M. fortuitum* into the extracellular milieu, promoting the production of extracellular cords.

Multiple hypotheses may explain why high doses of *M. fortuitum* are required to induce efficient and rapid killing of zebrafish larvae, as compared to *M. marinum* (Volkman et al., 2004; Davis and Ramakrishnan, 2009). One plausible explanation could be attributed to the fact that *M. fortuitum* remains phagolysosomal, presumably because of the lack of ESAT-6 secretion (Houben et al., 2012), although pan-genomic analyses seem to indicate that the *M. fortuitum* genome possesses an ESX-1 cluster (Dumas et al., 2016). Indeed, a clear link between translocation and mycobacterial virulence has been proposed, relying on a functional ESX-1 system and ESAT-6 secretion, which facilitates translocation of members of the *M. tuberculosis* complex and *M. marinum* from the phagolysosome to the cytosol (Houben et al., 2012). However, *M. abscessus* which escapes the phagosome lacks ESX-1, but it has been postulated that communication with the cytosolic compartment is favored by the presence of a functional ESX-4 secretion system (Roux et al., 2016; Laencina et al., 2018; Johansen et al., 2020). Future work is therefore required to investigate the contribution of the different ESX systems in the pathogenicity of *M. fortuitum*.

Moreover, we have harnessed the CFTR zebrafish model of infection as an innovative vertebrate recapitulating aspects of CF immuno-pathogenesis (Bernut et al., 2019). *M. fortuitum-*infected CFTR-depleted zebrafish rapidly succumbed to infection, reflecting a hypersusceptibility phenotype to this mycobacterial species in CF and providing a first glance into the vulnerability of CF patients to *M. fortuitum* infection. Previous work indicated CFTR participates in neutrophil chemotaxis to the infected sites and the adjustment of oxidative host defenses, conditioning efficient phagocyte-mediated bacterial killing, together generating a protective granulomatous response (Bernut et al., 2019). However, the role of neutrophils in the defense against *M. fortuitum* remains to be investigated in future studies. CFTR depletion is also associated with a deficiency in radical oxygen species production altering phagocyte-mediated killing. It is, therefore, very likely that this low oxidative response in *cftr* morphants results in increased intracellular loads of *M. fortuitum* and premature cell death. Thus, the reduced number of protective granulomas together with the uncontrolled extracellular mycobacterial spread leads to acute infection and larval death in *cftr* morphants. Our study indicates that *CFTR* is a regulator of host immunity to *M. fortuitum*, as suggested previously for *M. abscessus*. Importantly, CFTR depletion has been shown to have no impact on the control of host immunity to the non-pathogenic *M. smegmatis* nor to the pathogenic *M. marinum* (Bernut et al., 2019). These findings imply that species-specific restriction mechanisms occur for various NTM, an interesting observation that will require further investigation. Overall, this new animal model allows us to propose that *cftr* represents a gene of susceptibility to *M. fortuitum* infection.

This study also paves the way to new translational possibilities in the fight against *M. fortuitum* infections. The zebrafish model described here, particularly conducive to spatiotemporal imaging of *M. fortuitum* infections, may also be exploited to test the *in vivo* efficacy of known antibiotics or new drug treatments. It may represent a unique biological system allowing non-invasive observations to evaluate, in real time, the efficacy of compounds in a living vertebrate, as shown previously for *M. marinum* (Takaki et al., 2012) and *M. abscessus* (Bernut et al., 2014b; Dubée et al., 2015; Dupont et al., 2017; Raynaud et al., 2019) and applied to high-throughput *in vivo* testing of drug efficacy (Carvalho et al., 2011).

## Data Availability Statement

The raw data supporting the conclusions of this article will be made available by the authors, without undue reservation, to any qualified researcher.

## Ethics Statement

The animal study was reviewed and approved the Comité d'Ethique pour l'Expérimentation Animale de la Région Languedoc Roussillon under the reference CEEALR36-1145.

## Author Contributions

MJ conducted experiments, analyzed the data, and wrote the paper. LK conceived the idea of the project, analyzed the data, and wrote the paper. All authors contributed to the article and approved the submitted version.

## Conflict of Interest

The authors declare that the research was conducted in the absence of any commercial or financial relationships that could be construed as a potential conflict of interest.
